# Proteogenomic Reassessment Provides Novel Insight into the Life Cycle of *Tetrahymenathermophila*

**DOI:** 10.1016/j.mcpro.2025.101081

**Published:** 2025-09-30

**Authors:** Chen Gu, Mingkun Yang, Jing Zhang, Guangying Wang, Lu Fu, Kai Chen, Lujuan Li, Peng Zhang, Shuai Luo, Fangdian Yang, Jiao Zhan, Wei Miao, Feng Ge, Jie Xiong

**Affiliations:** 1 Key Laboratory of Aquatic Biodiversity and Conservation, Institute of Hydrobiology, Chinese Academy of Sciences, Wuhan, China; 2 University of Chinese Academy of Sciences, Beijing, China; 3 Key Laboratory of Algal Biology, Institute of Hydrobiology, Chinese Academy of Sciences, Wuhan, China; 4 Key Laboratory of Breeding Biotechnology and Sustainable Aquaculture, Institute of Hydrobiology, Chinese Academy of Sciences, Wuhan, China; 5 Key Laboratory of Lake and Watershed Science for Water Security, Chinese Academy of Sciences, Nanjing, China

**Keywords:** *T. thermophila*, proteogenomics, mass spectrometry, genome assessment, post-translational modification

## Abstract

*Tetrahymena thermophila* (*T. thermophila*), a well-established model organism, has been instrumental in advancing our understanding of evolutionarily conserved biological processes. A key biological feature of this unicellular eukaryote is its life cycle strategy, marked by three major stages: growth, starvation, and conjugation. Despite its prominence as a model system, functional genomic studies of *T. thermophila* have been constrained by limitations in the accuracy and completeness of gene discovery since the initial genome assembly in 2006. To address this gap, we performed a multi-stage proteogenomic analysis, combining genomic sequencing with high-resolution mass spectrometry (MS)-based proteomic profiling across 10 strategically selected life cycle states. This integrative approach enabled a comprehensive reassessment of gene discovery, leading to the validation of 24,319 previously predicted protein-coding genes and the identification of 383 novel genes. Additionally, our investigation systematically identified a diverse repertoire of post-translational modifications (PTMs), including 7123 modification sites distributed across 4705 proteins. These PTMs are postulated to exert critical regulatory functions during developmental phase transitions. Collectively, this work not only refines the *T. thermophila* gene catalog and enhances its utility as a robust genetic toolkit for advancing biological research but also offers new mechanistic insights into the molecular regulation of its life cycle progression.

## Highlights

• Proteogenomics integrates T2T genome, transcriptome, and proteome of *T. thermophila*.
• Protein expression mapped across 10 distinct life cycle states.
• 24,319 genes validated, 383 novel genes and 7123 PTM sites identified.
• Dynamics of protein expression and PTMs underpin life cycle regulation studies.
• Valuable resource for ciliate biology and cross-species comparative research.

## Graphical Abstract

**Figure:**
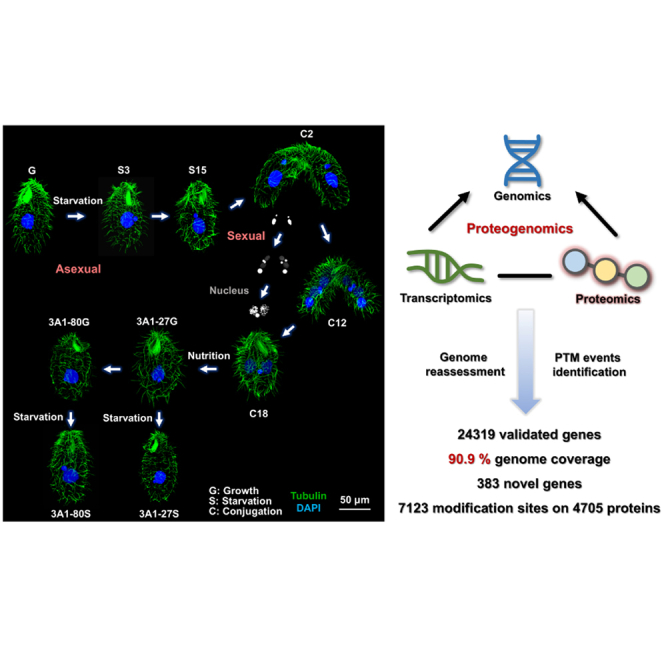

## In Brief

This study presents a comprehensive proteogenomic analysis of *Tetrahymena thermophila* across 10 life cycle states. By integrating high-resolution mass spectrometry with a telomere-to-telomere genome assembly, we validated over 24,319 predicted genes and discovered 383 novel genes. Additionally, we mapped 7123 post-translational modification sites, revealing dynamic regulatory mechanisms linked to development and environmental response. This work provides a valuable resource for advancing functional genomics and understanding molecular regulation in this key model organism.

The selection of an appropriate model organism is critical for advancing our understanding of cellular and molecular mechanisms. *Tetrahymena thermophila*, a well-established model protozoan, has emerged as an indispensable system for elucidating fundamental biological processes, attributed to its distinctive biological characteristics and experimental versatility (1). Despite being unicellular, it exhibits many core eukaryotic processes that are conserved in multicellular organisms (including humans) but are absent in other unicellular models such as *Saccharomyces cerevisiae* and *Schizosaccharomyces pombe* (2). To date, *T. thermophila* has contributed to numerous breakthroughs in molecular biology, including the identification of the first dynein (3), the characterization of telomeric structures and telomerase (4, 5), the discovery of catalytic RNA (6), and the elucidation of histone acetylation (7). Beyond its traditional roles, *T. thermophila* has become increasingly valuable in ecology and toxicology. Its central role in aquatic trophic networks has prompted investigations into interspecific interactions, particularly with prey bacterial and phages (8, 9). Furthermore, this organism displays both a high sensitivity to environmental toxins and a remarkable tolerance to heavy metals such as mercury (Hg) and cadmium (Cd), making it a valuable model for toxicological studies (10).

*T. thermophila* exhibits nuclear dimorphism, featuring a transcriptionally active somatic macronucleus (MAC) that governs cellular functions and a transcriptionally silent germline micronucleus (MIC) dedicated to genetic information preservation. This dual-nuclear organization enables *T. thermophila* to adapt effectively to complex and fluctuating environmental conditions (11). During nutrient abundance, the organism reproduces asexually through binary fission. However, under nutrient deprivation, it transitions to sexual reproduction via conjugation, during which the MIC undergoes meiosis to produce exchangeable gametes that recombine to form a zygotic nucleus (12). Following conjugation, the zygotic nucleus divides into four new MICs, two of which undergo extensive differentiation to develop into new MACs. This process involves the precise elimination of internal eliminated sequences (IESs), chromosome fragmentation, and telomere addition (11, 13). The sexual progeny produced require approximately 65 consecutive cell divisions to reach sexual maturity and restore mating competence (14). Despite advancements in the field, our understanding of the molecular mechanisms of the life cycle of *T. thermophila* is still fragmentary.

In 2006, the MAC genome of *T. thermophila* was first decoded by Sanger sequencing, a milestone that greatly accelerated research using the *Tetrahymena* model system (2, 15). With the development of ultra–high-coverage Nanopore technology, a complete telomere-to-telomere MAC assembly was achieved in 2021 (16). As for the MIC with more complex genome architecture, Sanger sequencing was carried out in 2011, but a full assembly was not available until 2016 (17, 18). The diploid MIC genome, comprising five centromere-bearing chromosomes totaling ∼157 Mb (17), contrasts sharply with the highly polyploid MAC genome. The latter lacks centromeres, consists of 181 chromosomes spanning ∼104 Mb, and exhibits ∼90-fold ploidy (19), with protein-coding regions constituting 48% of its sequence (2, 20). Despite these advances in genome sequencing and assembly, accurately identifying the full complement of protein-coding genes in *T. thermophila* remains a significant challenge. Contemporary bioinformatic pipelines typically combine *ab initio* prediction, homology-based inference, and structural feature detection to infer gene models (21, 22). However, these model-driven approaches are inherently prone to error, often generating incomplete or inaccurate predictions. For instance, conventional gene prediction algorithms prioritize open reading frames (ORFs) exceeding 100 codons, systematically overlooking small ORFs (sORFs) with validated coding potential (23, 24). Algorithmic limitations—including excessive reliance on homology-based comparisons and parameter biases in predictive models—further propagate inaccuracies, undermining the reliability of gene predictions (25, 26). These issues are exacerbated by eukaryotic genomic complexities such as pervasive repetitive elements, structural variations, and non-canonical promoter usage (27, 28). Moreover, dynamic regulatory mechanisms, including condition-specific gene expression, are poorly captured by traditional gene prediction workflows. Conventional pipelines depend on static datasets generated under restricted experimental conditions, limiting their capacity to elucidate context-dependent gene functions in fluctuating environments (29). Additionally, the lack of comprehensive post-translational modification (PTM) data hinders a holistic understanding of gene product functionality (30). Consequently, a comprehensive reassessment of gene discovery in *T. thermophila* is imperative to systematically elucidate the molecular and cellular mechanisms governing this model protozoan’s life cycle.

Proteomics provides a robust framework for validating predicted protein-coding genes. Innovations in mass spectrometry (MS) technology have enabled researchers to obtain robust experimental evidence at the proteomic level, thereby improving the depth and reliability of protein characterization (31, 32, 33). The emerging field of proteogenomics integrates MS-derived proteomic data to reassess gene discovery, facilitating the identification of previously unannotated genes (34, 35). By incorporating proteomic evidence into gene-level assessments, proteogenomics has contributed to the refinement of gene catalogs across a wide range of organisms (36, 37, 38, 39). Recent advances in high-resolution MS technologies, coupled with access to comprehensive genomic and transcriptomic datasets, have further propelled proteogenomics research forward (40, 41, 42, 43). Our prior investigations involving the model cyanobacterium *Synechococcus* sp. PCC 7002 and the model diatom *Phaeodactylum tricornutum* have demonstrated the efficacy of proteogenomic approaches in assessing both prokaryotic and eukaryotic gene discovery (44, 45).

To reassess gene discovery in *T. thermophila*, we conducted a proteogenomic analysis using high-quality MS data from 10 states in the life cycle of this model protozoan. This approach unequivocally identified numerous known genes as well as previously unannotated coding sequences within the *T. thermophila* genome. Furthermore, characterization of PTMs at the proteomic level, using the same MS dataset, revealed diverse PTM types that may serve critical regulatory functions during distinct life cycle states. These findings not only provide essential information for refining the gene catalog of *T. thermophila* but also offer novel insights into the regulatory dynamics governing its life cycle. Consequently, our proteogenomic strategy represents a significant advancement in the systematic understanding of *T. thermophila*'s molecular and cellular processes, thereby enhancing its utility as a model system in biological research.

## Experimental procedures

### Experimental Design and Statistical Rationale

To achieve in-depth protein coverage in this study, our sample collection spanned three major life cycle stages of *T. thermophila*: growth, starvation, and conjugation. Cells from 10 distinct states were harvested, and total protein extracts were prepared with modifications to the previously described protocol (44). Protein extracts from each state were separately subjected to in-gel tryptic digestion. Furthermore, the lysates were also processed with in-solution tryptic digestion and pre-fractionated on a TechMate C18 column using an UltiMate 3000 HPLC system. All peptides were analyzed on a Q Exactive HFX mass spectrometer. Proteomic data for each state were consolidated by integrating the results from both in-gel and in-solution digestion protocols. Peptides, expressed proteins, modified proteins and novel events were identified using the GAPE software integrated with the pFind search engine in open search mode (46, 47). Functional annotations of the identified *T. thermophila* proteins were performed based on KOG and GO terms, and the subcellular localization was analyzed by CELLO web tool (48). Conservation analysis was carried out by using reciprocal BLAST (49). Orthologous genes of novel proteins were identified using BLASTP against the UniRef database (50). RNA sequencing (RNA-seq) reads were aligned to the reference genome with TopHat v2.0.8 (51). Gene expression was quantified as fragments per kilobase of exon per million mapped fragments (FPKM) using a custom Perl script, and protein abundance was determined by normalized spectral abundance factor (NSAF) (52). The cluster analysis was performed by Cluster 3.0 and visualized by TreeView (53). The Pearson correlation coefficient was used to evaluate the association between transcriptomic and proteomic expression levels (54). In addition, Western blot analysis was performed to verify the widespread presence of methylation modifications in total protein samples. R scripts and Excel were used for the statistical analyses.

### Sample Preparation of *T. thermophila*

Wild-type *T. thermophila* strains CU428 (mating type VII) and SB210 (mating type VI) were cultured in super proteose peptone (SPP) medium (1% proteose peptone, 0.2% glucose, 0.1% yeast extract, 0.003% sequestrene) at 30 °C (55). For starvation, they were washed twice and cultured in 10 mM Tris-HCl (pH 7.4) at 30 °C (56). Conjugation was induced by starving CU428 and SB210 strains for 20 h before mixing equal volumes of the two strains. For each sample, 30 ml of cell suspension (∼3 × 10^5^ cells/ml) was centrifuged at 1500 rpm, and cells under vegetative growth were washed twice with 10 mM Tris-HCl (pH 7.4). The supernatant was discarded, and the cell pellet was immediately stored at −80 °C until further use. After 14 h co-culture, a single conjugating pair was isolated by mouth pipette, and one of its four sexual progeny was maintained in SPP medium by serial transfers. At ∼27 divisions (pre-sexual maturity) and ∼80 divisions (post-sexual maturity) cell samples were collected and cryopreserved in the same manner. For extraction, the frozen cell pellets were resuspended in phosphate-buffered saline (PBS) buffer (137 mM NaCl, 2.7 mM KCl, 8 mM Na_2_HPO_4_, 2 mM KH_2_PO_4_, pH 7.2) supplemented with protease and phosphatase inhibitor cocktails. The suspension was sonicated on ice with 3 s on/3 s off cycles for 5 min at 130 W (JY92-IIN, Ningbo Scientz Biotechnology Co., Ltd), then centrifuged at 10,000*g* for 10 min at 4 °C to remove insoluble debris. The clarified lysate was collected, and protein concentration was measured using a bicinchoninic acid (BCA) assay kit (P0012S, Beyotime Biotechnology) according to the manufacturer’s instructions. Aliquots were stored at −80 °C for downstream analyses.

### Protein Digestion and Liquid Chromatography–MS/MS Analysis

In-solution and in-gel digestions were performed with minor modifications to established protocols (44, 57). For in-solution digestion, proteins were reduced with 25 mM DTT for 40 min at 37 °C, then alkylated with 50 mM iodoacetamide (IAA) for 10 min at room temperature in darkness. Samples were finally digested with sequencing-grade trypsin (Promega) at a 1:100 enzyme-to-substrate ratio overnight at 37 °C. Peptides were fractionated using an UltiMate 3000 HPLC system (Thermo Fisher Scientific Corporation) with a TechMate C18 column (2.0 mm × 150 mm, 5 μm particle size). Peptides were washed with a gradient of buffer B (10 mM NH_4_OH in 80% acetonitrile) 5% to 80% at a rate of 0.2 ml/min for 80 min. Finally, the eluted peptides were combined into 10 fractions and dried. For in-gel digestion, proteins were resolved on a 12% SDS-PAGE gel, and each lane was cut into 10 gel slices. Gel pieces were destained with 35% acetonitrile and 50 mM NH_4_HCO_3_, followed by reduction with 25 mM DDT and alkylation with 50 mM IAA. Protein was finally digested with trypsin (1:100 w/w) (Promega) overnight at 37 °C. Peptides were extracted twice with extraction buffer (67% [vol/vol] acetonitrile, 5% [vol/vol] trifluoroacetic acid). The peptide supernatants and extracts were combined, desalted, and completely dried.

All peptides were resolved in 0.1% formic acid (solvent A) and separated on an EASY-nLC 1200 nano-LC system with a 15 cm × 75 μm C18 column (Thermo Fisher Scientific). Peptides were eluted using solvent B (0.1% formic acid in 80% acetonitrile) at 300 nl/min with a 125-min solvent gradient: 0 to 1 min, 2 to 6% B; 1 to 61 min, 6 to 17% B; 61 to 86 min, 17 to 23% B; 86 to 105 min, 23 to 32% B; 105 to 115 min, 32 to 38% B; 115 to 116 min, 38 to 90% B; 116 to 125 min, 90% B. Eluting peptides were analyzed on a Q Exactive HFX mass spectrometer (Thermo Fisher Scientific). Data were acquired in data-dependent acquisition (DDA) mode using Xcalibur 3.0, with each cycle consisting of one high-resolution full MS scan (m/z 350–1800 at 60,000 resolution) followed by MS/MS of the top 20 most intense precursor ions (charge states 2–6). Precursors were isolated with a 1.6 m/z window and fragmented by HCD at 28 normalized collision energy. The electrospray voltage was set to 2.2 kV, dynamic exclusion for selected precursors was 30 s, and maximum ion injection times were 30 ms for MS scans and 50 ms for MS/MS scans.

### Proteogenomic Analysis

Raw MS data were converted to MGF format by MSConvert tool in ProteoWizard software (version 3.0.4416) and submitted to the GAPE software for peptide and protein identification (44). First, the telomere-to-telomere MIC reference genome was constructed using the publicly available National Center for Biotechnology Information (NCBI) genome assembly (GCA_016584475.1), following a previously described strategy (16), and is publicly available in the genome warehouse of the National Genomics Data Center (Project ID: PRJCA042635, https://ngdc.cncb.ac.cn/). The *T. thermophila* protein reference database (*Tetrahymena*_*thermophila*_protein_sequences_v5) was downloaded from *Tetrahymena* genome database (TGD, http://www.ciliate.org). Then, a customizable proteogenomic database was generated by performing six-frame translation of the genome and assembling RNA-seq reads using Trinity with default parameters to reconstruct the authentically expressed transcripts. The assembled transcripts were subsequently translated in three frames to generate protein sequences (58). Only sequences ≥20 amino acids were retained and common contaminants (trypsin, human keratins) were appended. Finally, all MS/MS data were then searched against the proteogenomic database (10,346,819 protein entries) and protein reference database (26,742 entries) using the pFind search engine (version 3.2) incorporated into GAPE software (44, 46). Up to two missed cleavages were permitted in the search, with mass tolerances for precursor and fragment ions both specified as 20 ppm. An open search strategy was applied without the need to predefine modification parameters, thereby facilitating unbiased identification of both known and unknown peptide modifications (47). Minimum peptide length was set at six while the estimated false discovery rate (FDR) threshold for peptide and protein was specified at maximum 1%. The peptides matching only the customizable proteogenomic database were designated genome search-specific peptides (GSSPs) and novel coding sequences were predicted by GAPE.

### Functional Annotation and Bioinformatics Analysis

Functional annotations of the identified *T. thermophila* proteins were performed based on KOG (eukaryotic orthologous group) and GO (gene ontology) terms. Protein structural domains were queried against the Pfam and InterPro databases for domain-specific annotations. Conservation analysis of *T. thermophila* proteins was conducted using reciprocal BLAST following the methodology outlined by Rastogi *et al*. (49). Orthologous genes of these novel proteins were identified using BLASTP against the UniRef database (50), as described by (45). The genomic locations of identified peptides and proteins in *T. thermophila* were visualized using Circos software (59). All statistical analyses were performed using R software (60).

### RNA and Protein Expression Analysis

Total RNA was extracted using the RNeasy Protect Cell Mini Kit (Qiagen) from vegetative growth cells at 24, 48, and 72 h cultivation, as well as from sexual progeny 3A1 (CU428 × SB210) collected at ∼27 divisions and ∼80 divisions under vegetative growth and starvation respectively. The RNA was sequenced and mapped as described previously (61). All raw RNA-seq datasets generated in this study have been deposited in National Genomics Data Center, China National Centre for Bioinformation/Beijing Institute of Genomics, Chinese Academy of Sciences (PRJCA039481, https://ngdc.cncb.ac.cn/). The C2-state RNA data were obtained from Zhang *et al*. (62), and the S3-state RNA data from Xiong *et al.* (63). After filtering the low-quality RNA-seq reads, the remaining reads were mapped to the *T. thermophila* genome using TopHat (version 2.0.8) (51). Gene expression levels were quantified as FPKM using a custom Perl script. Protein expression levels were quantified using the NSAF (52). Both RNA FPKM and protein NSAF data were normalized using Z-score transformation to ensure comparability across samples. Heatmaps for hierarchical clustering and visualization were created with Cluster 3.0 and TreeView (53). Transcriptomic data under Hg and Cd exposure were produced following the same procedures. Log_2_-fold change (log_2_FC) in expression levels were calculated by comparing treated samples with untreated growth controls. Heatmaps for this dataset were constructed using Origin 2018 (64).

### Western Blot Analysis

*T. thermophila* whole-cell extracts were prepared as detailed in the ‘Sample Preparation of *T. thermophila’* section. Western blotting analysis was conducted following the protocol described by Chen *et al*. (65). The primary antibodies (anti-mono/di-methyllysine and anti-trimethyllysine, PTM-602 and PTM-601; Jingjie PTM BioLab) were used at a 1:1000 dilution, and the secondary antibody (HRP-goat anti-rabbit IgG, PTM-6261; Jingjie PTM BioLab) was used at a 1:2000 dilution. GAPDH (glyceraldehyde-3-phosphate dehydrogenase) was used as the control (HRP-60004, ProteinTech, 1:10,000 dilution).

### Fluorescent Staining of Tubulin and Nucleus

Ciliary and nuclear staining of *T. thermophila* cells were carried out using an improved method described by Pan *et al*. (66). Cells were incubated with Tubulin-Atto 488 fluorescent dye (Z4044, Singularity Fluorescence Nanjing Biotechnology Co., LTD) diluted 1:20 in PBS buffer. Images were acquired with a Leica TCS SP8 laser scanning confocal system using 405 nm and 488 nm excitation wavelengths for DAPI and tubulin, respectively, and analyzed with Leica LAS AF Lite software.

## Results

### Proteomic Data Generation Across *T. thermophila* Life Cycle

**Fig. 1:**
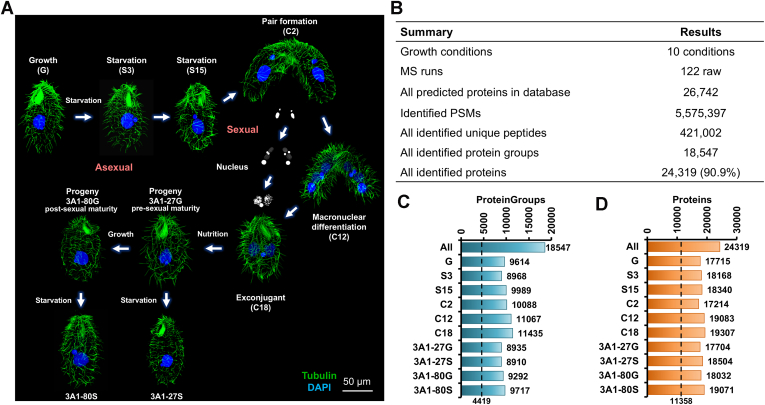
**Overview of the Proteogenomic Results.***A*, schematic representation of the life cycle sampling of *T. thermophila* in this study. The sampling included 10 distinct states: Vegetative growth (G); Starvation for 3 h (S3); Starvation for 15 h (S15); Conjugation for 2 h (C2); Conjugation for 12 h (C12); Conjugation for 18 h (C18); Sexual progeny after ∼27 divisions under nutrient-rich conditions (3A1-27G); Sexual progeny after ∼27 divisions under starvation conditions (3A1-27S); Sexual progeny after ∼80 divisions under nutrient-rich conditions (3A1-80G); Sexual progeny after ∼80 divisions under starvation conditions (3A1-80S). Cilia were labeled with Tubulin-Atto 488 fluorescent dye (*green*), and nuclei were marked with DAPI (*blue*). *B*, overall summary of proteome generation and protein identifications in the present study. *C*, the number of protein groups identified in each sample and cumulatively. The dashed line represents the conserved protein groups shared by all samples. *D*, the number of proteins identified in each sample and cumulatively. The dashed line represents the conserved proteins shared by all samples.

**Supplementary Figure S1:**
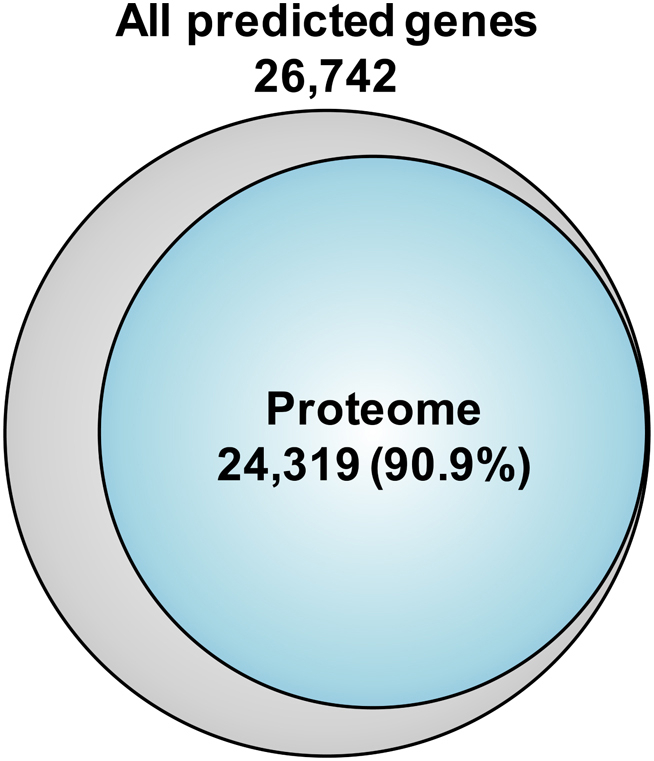


**Supplementary Figure S2:**
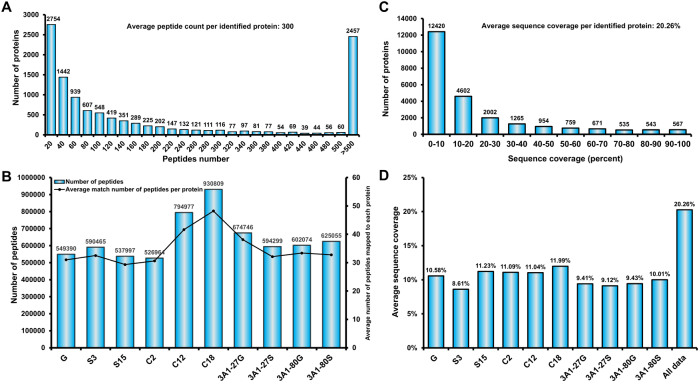


In this study, we collected 10 distinct samples to comprehensively capture the proteomic landscape across the life cycle of *T. thermophila*. These states encompass vegetative growth, starvation-induced phase, conjugation, as well as the pre- and post-sexual maturity states of sexual progeny (Fig. 1*A*). By applying a stringent false discovery rate (FDR) threshold (FDR ≤1%), we identified a total of 421,002 unique peptides across 122 raw MS runs (Fig. 1*B*). Mapping these peptides to the reference genome database validated the expression of 24,319 protein-coding genes (18,547 protein groups), representing 90.9% of the genes predicted by the genomic database (Supplemental Fig. S1). All identified peptides and proteins in this study are available at https://iprox.cn.org with the identifier IPX0011857000 (under the file name “List of the proteomic identification in TM”). To assess peptide distribution and sequence coverage, we quantified the number of peptides and amino acids mapped to each protein (Supplemental Table S1). On average, each identified protein was supported by 300 mapped peptides in total (including redundant peptides), with 2457 proteins matched by more than 500 peptides (Supplemental Fig. S2*A*). The average number of unique peptides per protein was 17.3. Furthermore, the number of peptides identified per sample ranged from 526,964 to 930,809, with C12 (794,977 peptides) and C18 (930,809 peptides) showing the highest counts (Supplemental Fig. S2*B*). C12 and C18 also exhibited the highest average number of matched peptides per protein, ranging from 40 to 50. These results suggest that during MAC development and the exconjugant states following mating-pair separation, cells undergo extensive DNA rearrangements and progressive establishment of transcriptional and translational machinery in the new MAC, accompanied by heightened translational activity, which likely accounts for the increased protein abundance. Based on the assumption that existing gene models are correctly annotated, the average sequence coverage per identified protein was 20.26% (Supplemental Fig. S2, *C* and *D*), with 567 proteins exhibiting 90 to 100% sequence coverage. Across samples, coverage varied modestly, ranging from 8.61% to 11.99%. Collectively, the depth and consistency of peptide and sequence coverage underscore the high quality of our proteome data, ensuring robust and trustworthy results.

**Supplementary Figure S3:**
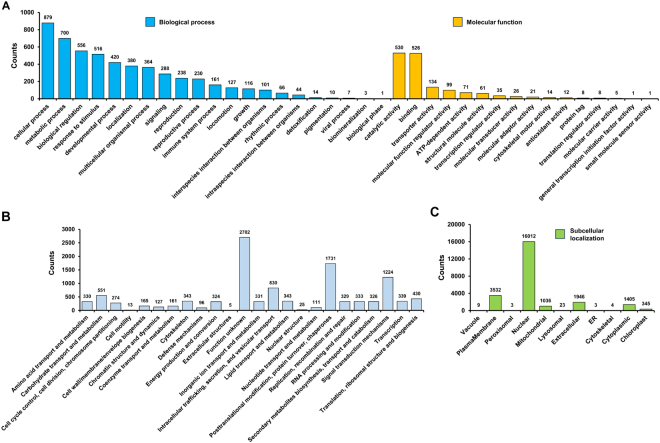


**Supplementary Figure S4:**
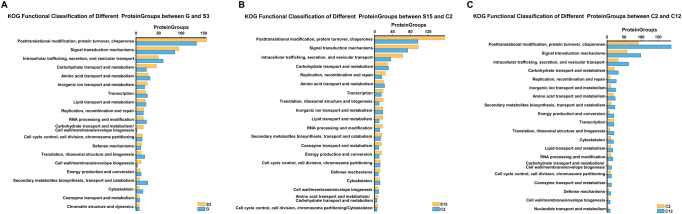


To systematically clarify the biological function of these identified proteins, we performed functional annotation in our dataset (Supplemental Table S2). GO classification revealed that these proteins are involved in a wide range of biological processes, including cellular processes, metabolic processes, biological regulation, response to stimuli, and developmental processes. Regarding molecular functions, the majority of the proteins are associated with catalytic activity, binding, and transporter activity (Supplemental Fig. S3*A*). Specifically, these proteins play functional roles in processes such as PTMs, protein turnover, signal transduction, intracellular trafficking, and vesicular transport, emphasizing their potential roles in maintaining cellular homeostasis, regulating signaling pathways, and supporting essential processes such as protein maturation, trafficking, and secretion (Supplemental Fig. S3*B*). Subcellular localization analysis revealed that the identified proteins were predominantly located in the nucleus (16,012), followed by plasma membrane (3532), extracellular areas (1946), cytoplasm (1405), mitochondria (1036), chloroplasts (345), and other regions (42) (Supplemental Fig. S3*C*). Differential proteins identified at various states (G *versus* S3, S15 *versus* C2, and C2 *versus* C12) exhibit functional characteristics that also converge on these core biological processes, such as PTM, protein turnover and signal transduction (Supplemental Fig. S4), further emphasizing their critical importance in *T. thermophila*.

**Supplementary Figure S5:**
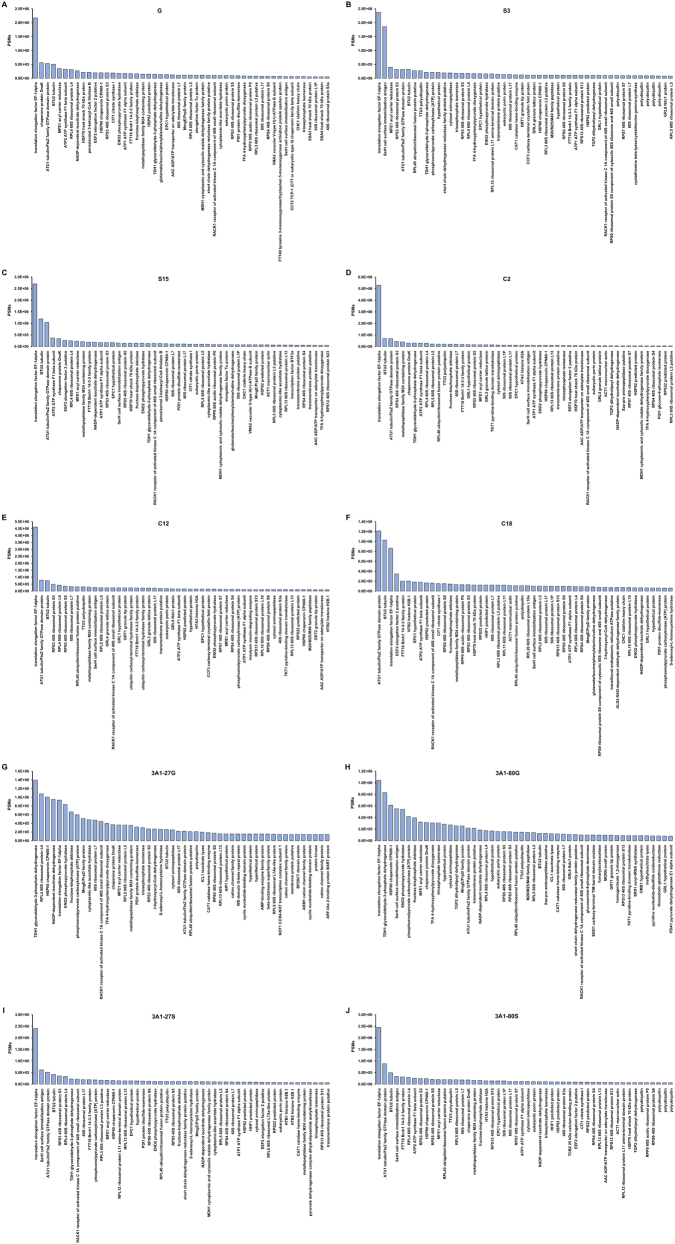


The distributions of identified proteins and protein groups across different samples are shown in Figure 1, *C* and *D*. Comparative analysis revealed 4419 protein groups, comprising 11,358 proteins that were consistently present among all samples. These conserved proteins are likely to play key biological roles throughout the life cycle of *T. thermophila*. Analysis of peptide–spectrum match (PSM) distributions for each sample (Supplemental Fig. S5) revealed the consistent presence of translation elongation factors, tubulins, ribosomal subunits, ATP synthases, and molecular chaperones across all life cycle states, underscoring their indispensable roles in protein synthesis, cellular dynamics, energy homeostasis, and protein folding (67, 68, 69, 70).

### Proteomic and Transcriptomic Comparison of *T. thermophila*

**Supplementary Figure S6:**
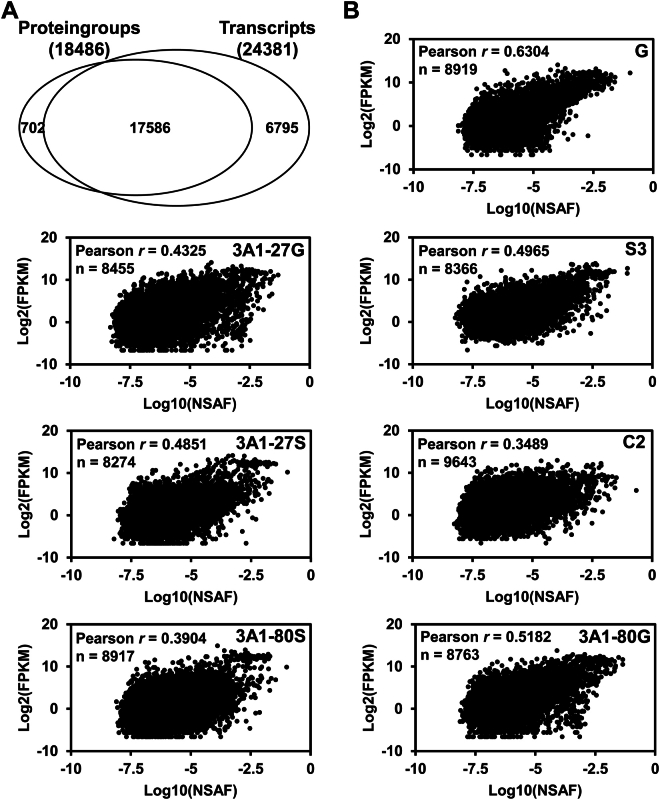


To further confirm the expression of these identified proteins, we performed transcriptome analyses and NSAF analyses of *T. thermophila* across different states of its life cycle. A total of 24,381 protein-coding transcripts were identified, surpassing the 18,486 protein groups predicted in the proteomic analysis (Supplemental Fig. S6*A*). Among these, 17,586 proteins were shared between transcriptomic and proteomic datasets, with 702 proteins exclusive to the proteome. The correlation coefficients between transcriptomic and proteomic data across different states revealed values ranging from 0.35 to 0.63 (Supplemental Fig. S6*B*), suggesting a moderate correlation between mRNA and protein levels. Similar correlation ranges have been reported in previous studies, emphasizing the need for proteomic data to complement gene annotation and functional characterization (71, 72, 73).

**Fig. 2:**
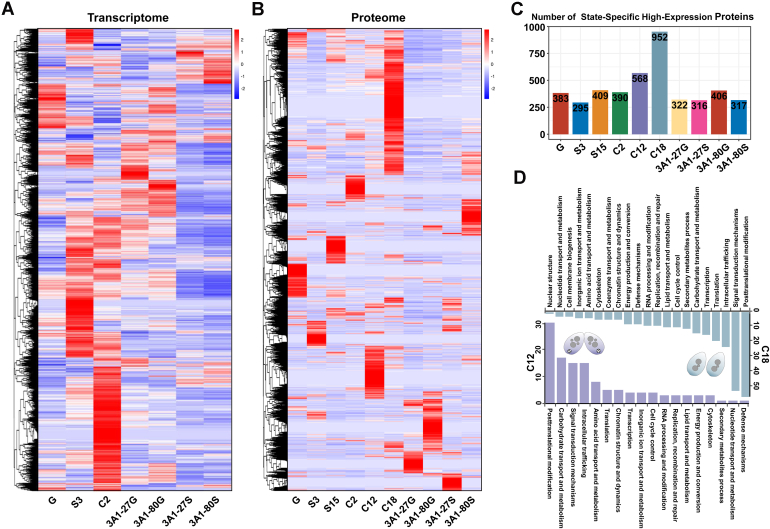
**Proteomic-Transcriptomic Expression Profiles and Identification of Highly Expressed Proteins in *T. thermophila*.***A*, clustering-based transcriptome heatmap of samples at 7 states, based on FPKM values of all identified proteins. *B*, clustering-based proteome heatmap of samples at 10 states, using NSAF to quantify relative protein abundance. The source data for Figure 2*A* and 2*B* are available in Supplemental Table S3. *C*, number of state-specific high-expression proteins. Proteins were defined as state-specific high-expression if their normalized abundance (from proteomic data in Fig. 2*B*) was greater than 2 at 1 state and less than 0 at the remaining 9 states. *D*, KOG functional annotation of state-specific highly expressed proteins identified in C12 and C18, as shown in Fig. 2*C*.

Figure 2, *A* and *B* depict distinct transcriptomic and proteomic expression profiles across the life cycle states, each characterized by a unique signature. These observations suggest that the composition of proteins at each state may underpin the corresponding functional requirements. To probe this functional linkage, we identified state-specific high-expression proteins as those with normalized expression >2 in one state and <0 in all others (Fig. 2*C*). Notably, the highest number of state-specific high-expression proteins was observed at C12 and C18. In the conjugation cycle of *T. thermophila*, C12 (new MAC formation) and C18 (exconjugant) are critical windows of nuclear differentiation. These two states were accompanied by a substantial presence of highly expressed proteins, potentially linked to intricate nuclear remodeling and genome reorganization processes. KOG functional annotation (Fig. 2*D*) showed that at C12, PTM and carbohydrate metabolism proteins dominated and, together with factors in chromosome dynamics, DNA repair, intracellular trafficking, and signal transduction, drove nuclear restructuring for new MAC formation. At C18, the protein profile shifts to broad regulatory control: PTM and signal-transduction factors stay high, while translation, transcription and RNA-processing proteins rise to establish the new MAC’s transcriptional machinery. Concurrent increases in cell-cycle regulators and membrane-biogenesis proteins mark the move from conjugation to cell separation, and sustained DNA-repair activity underscores ongoing genomic surveillance during this late developmental state.

### Discovery of Global Post-Translational Modifications in *T. thermophila*

**Fig. 3:**
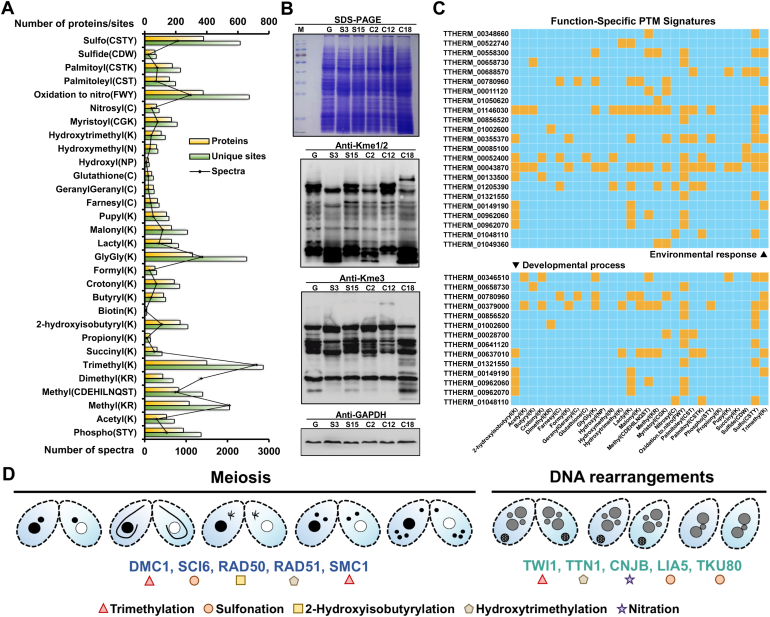
**Summary of Identified PTM Proteins in *T. thermophila*.***A*, distribution of the number of proteins, unique modification sites and spectra associated with different PTM types in *T. thermophila*. The source data for Fig. 3*A* is available in Supplemental Table S4. *B*, relative proteome-wide levels of methylation. Western blots were performed using antibodies specific for mono-/dimethyl-lysine and trimethyl-lysine. Coomassie brilliant *blue* staining (*top panel*) confirms equal total protein loading. GAPDH was used as an additional control. *C*, function-specific PTM signatures. PTM profiles of proteins related to environmental response and developmental processes. Orange squares indicate the presence of specific PTM types on corresponding proteins. *D*, PTMs of proteins involved in meiosis and DNA rearrangement events during sexual reproduction in *T. thermophila*.

The KOG functional annotation of all identified proteins (Supplemental Fig. S3*B*) and the functional comparison of differentially expressed proteins across various states (Supplemental Fig. S4) indicate that PTMs play an essential role throughout the entire life cycle of *T. thermophila*. Based on the open-search results of our MS data, we can directly count the types of PTMs on each protein. Here, we focused on 30 common eukaryotic modifications and identified 4705 modified proteins with 7123 modification sites by using a previous reported search strategy (Fig. 3*A* & Supplemental Table S4). Detailed information on the PTM search is provided in Supplemental Table S5. Among these identified PTMs, lysine methylation modifications, including mono/di-methylation (Kme1/2) and trimethylation (Kme3), exhibited the highest abundance levels. To validate the presence of these modifications, we performed Western blot analysis of total proteins isolated from six different states using antibodies specific for Kme1/2 and Kme3. The results revealed state-specific variations in modification levels, which may reflect differences in functional roles (Fig. 3*B*).

**Supplementary Figure S7:**
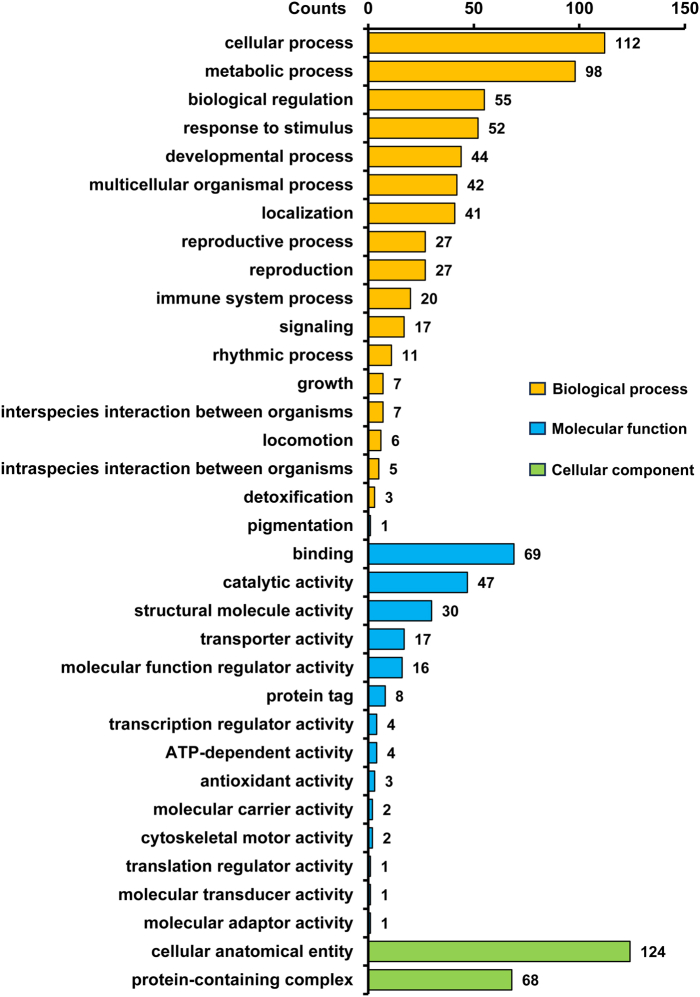


**Supplementary Figure S8:**
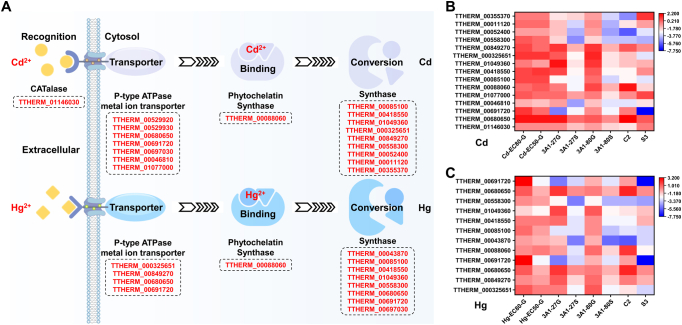


We further explored the function of modified proteins by performing GO functional annotation based on their biological processes, molecular functions, and cellular components (Supplemental Table S6). As shown in Supplemental Fig. S7, the analysis indicates that these proteins participate in a broad range of biological processes—such as cellular, metabolic, regulatory, stimulus response, and developmental activities. In terms of molecular function, binding activities (e.g., ATP, protein, metal ion and nucleic acid binding) dominate, followed by catalytic activities, while their localization spans diverse cellular components. Environmental response and developmental process are critical for *T. thermophila* during its life cycle. After sensing environmental nutrient scarcity, *T. thermophila* initiates conjugation and undergoes a series of developmental events that culminate in the production of sexual progeny. Our findings indicate that proteins involved in both developmental process and environmental response exhibit a wide range of PTMs, including acetylation, phosphorylation, and trimethylation (Fig. 3*C*). The conjugation process in *T. thermophila* has been extensively studied due to its characteristic nuclear differentiation events, such as MIC meiosis, MAC development, and cytoplasmic inheritance, highlighting their significance as critical processes driving genetic reorganization during sexual reproduction. In our dataset, meiosis-related proteins (DMC1, SCI6, RAD50, RAD51 and SMC1) and DNA-rearrangement–associated proteins (TWI1, TTN1, CNJB, LIA5 and TKU80) were found to bear PTMs (Fig. 3*D*), which likely modulate their functional activities. In addition, further analysis of the modified proteins involved in environmental response revealed that a substantial subset is associated with heavy metal metabolism (Supplemental Table S7). Heavy metals like Cd and Hg, known for their toxicity and bioaccumulation (74, 75), pose major environmental risks. Leveraging our proteome data, we identified 11 Hg- and 18 Cd-related genes (Supplemental Fig. S8*A*), encoding proteins involved in metal recognition, transport, binding and conversion. Transcriptomic upregulation under stress underscores their role in homeostasis, positioning *T. thermophila* as a model for bioremediation strategies (Supplemental Fig. S8, *B* and *C*).

### Novel Gene Identification in the *T. thermophila* Proteome

**Fig. 4:**
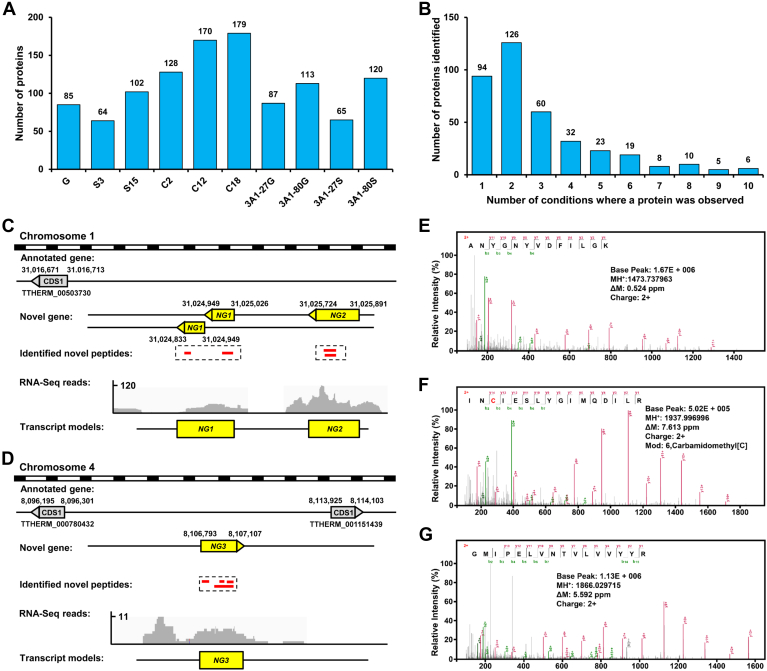
**Identification of Novel Genes in *T. thermophila*.***A*, number of newly identified proteins detected at each sample. *B*, distribution of novel proteins across 1 to 10 samples. The x-axis indicates the number of samples in which each protein was detected. *C*, novel peptides mapping to non-coding regions. Four peptides were mapped to two regions on chromosome one of the *T. thermophila* genome that lacks annotated genes. *D*, novel peptides mapping to intergenic regions. Four peptides were mapped to a region on chromosome four of the *T. thermophila* genome that lacks annotated genes. *E*-*G*, the MS spectra of three novel peptides (from NG1–NG3) identified by proteogenomic analysis. The peptides shown in *E* and *F* (NG1 and NG2, respectively) originate from the non-coding regions highlighted in *C*, whereas the peptide in *G* (NG3) maps to the intergenic region depicted in *D*.

**Supplementary Figure S9:**
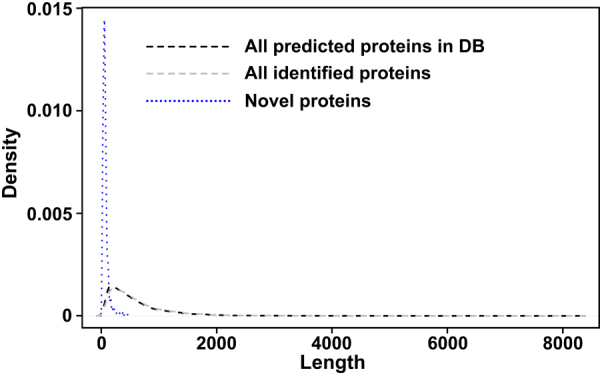


**Supplementary Figure S10:**
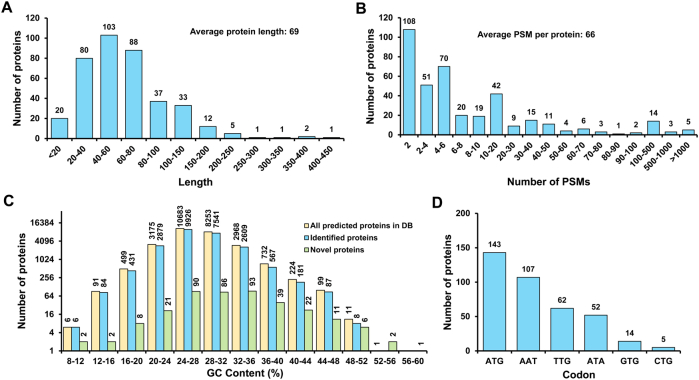


To identify potential novel protein-coding genes in *T. thermophila*, we excluded peptides that mapped to existing protein database entries and performed *de novo* gene predictions with GSSPs by using the developed GAPE pipeline (44). Proteomic analysis across various life cycle states identified distinct numbers of previously unannotated novel genes within each sample (Fig. 4*A*). A total of 383 novel genes were detected with at least two unique GSSPs (Supplemental Table S8). These novel proteins are significantly shorter than both the identified proteins and those predicted in the genomic database, with an average length of 69 amino acids (Supplemental Figs. S9 and S10*A*). Each protein had an average PSM of 66 (Supplemental Fig. S10*B*). Coding sequences of novel proteins exhibit a slightly higher GC content compared to those of genome-predicted proteins (Supplemental Fig. S10*C*). Interestingly, 94 genes were exclusively detected in one sample, implying potential state-specific expression and specialized functions associated with different phases of *T. thermophila*’s life cycle (Fig. 4*B*).

As shown in Figure 4*C*, we identified four unique intergenic peptides mapped to two specific regions on chromosome one of the *T. thermophila* genome that lack annotated genes, suggesting the presence of two novel protein-coding genes (*NG1* and *NG2*). Both *NG1* and *NG2* encode proteins harboring the conserved domain of unknown function 3638 (DUF3638), which is widely distributed among eukaryotic proteins. The evolutionary conservation of this domain suggests a role in fundamental cellular processes, although its precise molecular mechanisms remain undefined (76). Additionally, four novel peptides mapped to intergenic regions on chromosome four were identified as a novel gene *NG3* (Fig. 4*D*). NG3 shows significant homology to dynein heavy chain 1 (DYNC1H1), a central component of the cytoplasmic dynein complex responsible for retrograde microtubule-based transport and implicated in intracellular vesicle trafficking, organelle positioning, and mitotic spindle assembly (77, 78). The discovery of these genes was also supported by RNA-seq data. Figure 4, *E*–*G* showed the MS2 spectra of three novel peptides (from NG1–NG3), with a series of b- and y-ions.

Among the novel genes, besides of the typical initiation codons ATG, GTG, CTG, and TTG, we observed some novel genes bearing the noncanonical codons (AAT and ATA) as translation initiation codons (Supplemental Fig. S10*D*). ATG is the canonical initiation codon and predominates among newly identified genes. In contrast, GTG, CTG, and TTG can serve as alternative start codons and are associated with specific regulatory mechanisms of gene expression (79, 80, 81). The unconventional use of AAT and ATA as initiation codons may indicate the presence of unique translational mechanisms in *T. thermophila*. The observed codon usage bias suggests evolutionary pressures that may optimize translation efficiency and regulatory flexibility, as commonly seen in eukaryotes (82, 83, 84, 85). Comparative studies could reveal whether similar pressures influence codon usage in other protozoans or unicellular organisms.

### Comparison of Proteome and Transcriptome for Novel Genes

**Fig. 5:**
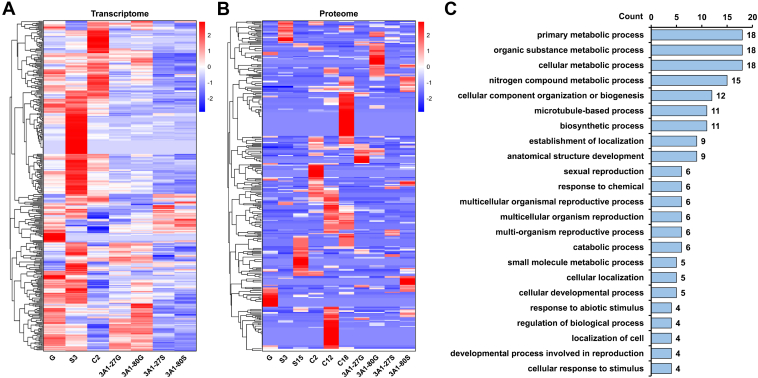
**Integrative Proteomic and Transcriptomic Profiling of Novel Genes.***A*, clustering-based transcriptome heatmap of samples at seven states, based on FPKM values of all identified novel proteins. *B*, clustering-based proteome heatmap of samples at 10 states, using NSAF to quantify relative abundance of identified novel proteins. The source data for Figure 5*A* and 5*B* are available in Supplemental Table S9. *C*, the number of identified novel genes annotated to GO biological process.

**Supplementary Figure S11:**
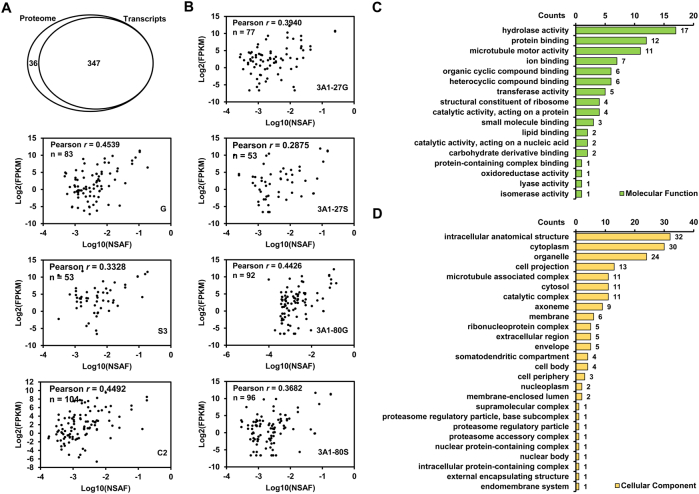


To further confirm the existence of the novel genes identified, we conducted transcriptome analyses using RNA-seq data from seven distinct life cycle states of *T. thermophila* (Supplemental Table S9). Of the 383 novel genes identified at the proteomic level, 347 were independently supported by transcriptomic data (Supplemental Fig. S11*A*), demonstrating the reliability of novel gene annotations. The absence of the remaining 36 novel genes in the transcriptome is likely due to the smaller sample size of the transcriptomic dataset, as well as the shorter lengths and lower expression levels of some of the newly identified genes. Correlation analysis revealed a moderate association between transcriptomic and proteomic expression data of the novel genes, with Pearson correlation coefficients ranging from 0.29 to 0.45 (Supplemental Fig. S11*B*). Moreover, the expression patterns of these novel genes varied markedly across different states (Fig. 5, *A* and *B*). The distinct expression profiles observed at both proteomic and transcriptomic levels underscore the potential functional roles of these novel genes. Based on GO classification, we performed a statistical analysis of the functions associated with these novel genes (Supplemental Table S10). In the “biological process” category, the novel gene are predicted to participate in a broad range of metabolic processes, including primary metabolic processes, organic substance metabolic processes, cellular metabolic processes, and nitrogen compound metabolic processes (Fig. 5*C*). In the “molecular function” category, the predominant functions include hydrolase activity, protein binding, and microtubule motor activity (Supplemental Fig. S11*C*). In addition, GO terms in the “cellular component” category reveal a widespread distribution (Supplemental Fig. S11*D*).

We then analyzed the differential expression patterns of these novel genes across distinct states from a functional perspective. During vegetative growth (G), highly expressed novel genes were enriched in proteolysis, peptide metabolism, and macromolecular metabolic processes, indicating active protein turnover to support sustained cell division (86). Under starvation (S), certain novel genes related to PTMs (e.g., ubiquitination) and lipid or fatty acid catabolism are upregulated, regulating protein degradation and lipid breakdown to maintain energy balance and cell survival under nutrient stress (87, 88). In early conjugation (C2), upregulated novel genes were enriched in RNA and protein biosynthesis pathways, consistent with increased transcriptional and translational activity during meiotic prophase (89). Upon entering the new MAC formation state (C12), the function of highly expressed novel genes shifts toward nucleotide metabolism, and their coordination with ATP biosynthesis may provide the substrates and energy required for extensive DNA rearrangements that characterize this state. During late conjugation (C18), the novel gene expression signature demonstrates a functional association with axonemal dynein complex assembly. This finding aligns with previous observations that *Tetrahymena* maintains ciliary regeneration capacity under starvation conditions (90). Our results further suggest that exconjugants may activate ciliary assembly machinery after separation of conjugating pairs.

### Functional Roles of State-specific Proteins in the *T. thermophila* Life Cycle

**Fig. 6:**
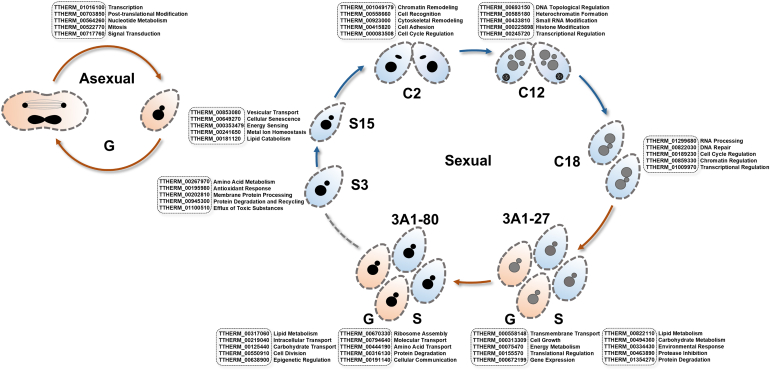
**State-specific proteinidentification andfunctional characterization across thelife cycle of *T. thermophila*.** For each state, five representative proteins exclusively identified in that state are listed with their IDs and annotated functional roles. *Orange* arrows and cells denote nutrient-rich culture conditions, while *blue* arrows and cells indicate nutrient-depleted conditions.

Functional annotations in this study reveal a high degree of adaptability between the biological functions of state-specific proteins in *T. thermophila* and the corresponding physiological demands (Fig. 6). During vegetative growth (G), the state-specific proteins involved in transcription, PTMs, nucleotide metabolism, and mitosis suggest that cells enhance genetic information transfer and proliferation to support rapid division. In the short-term starvation phase (S3), the activation of proteins associated with amino acid metabolism, antioxidant responses, and the efflux of toxic substances reflects a survival strategy in which cells maintain energy homeostasis and counteract oxidative damage through catabolic processes. As starvation progresses to the later state (S15), the regulation of energy sensing, lipid catabolism, and metal ion homeostasis further underscores adaptive resource reallocation under prolonged nutrient deprivation. During conjugation, state-specific protein functions exhibit a distinct temporal compartmentalization. The early state (C2) are marked by cytoskeletal remodeling and chromatin regulation—likely facilitating structural adjustments for conjugated pair formation and subsequent nuclear elongation. The mid-state (C12), characterized by the development of the new MAC, depends on DNA topological control and small RNA modifications to ensure the precision of genetic recombination and epigenetic reprogramming. In the later state (C18), the emphasis shifts to DNA repair and RNA processing, which may be critical for rectifying genome damage incurred during sexual reproduction. Moreover, state-specific protein functions vary among progeny at different sexual maturation states. Prior to sexual maturity (3A1-27S), the functions of specific protein were primarily associated with protein degradation and environmental stress response mechanisms. After sexual maturation (3A1–80S), the emergence of intercellular communication–related proteins may correlate with the recovery of mating competence under starvation.

## Discussion

This study presents a high-quality proteome dataset spanning 10 life cycle states of *T. thermophila*, offering protein-level validation for approximately 91% (24,319) of its annotated protein-coding genes. Through proteogenomic reassessment, 383 novel coding genes were identified, expanding the current gene catalog of this organism. Additionally, this work provides the first systematic exploration of PTMs in *T. thermophila*, uncovering their widespread functional roles across developmental stages. Together, these results establish the most comprehensive proteomic resource for this model organism to date.

Proteogenomics, which combines MS-based proteomics with multi-omics data, enables the comprehensive reassessment of gene discovery. Nevertheless, the assembly of a complete proteomic atlas persists as a formidable challenge, particularly in eukaryotes with intricate genomic architectures. It is reported that the achievable proteomic coverage in eukaryotic organisms is generally limited (91). For instance, reported coverage rates include *Pyrus bretschneideri* (41.9%), *Arabidopsis thaliana* (48%), and *Caenorhabditis elegans* (54%) (42, 92, 93). Even well-studied models such as *Drosophila melanogaster* (63%), *Saccharomyces cerevisiae* (83.5%), and humans (84%) exhibit significant undetected protein fractions (94, 95, 96). Notably, our analysis achieved 90.9% coverage for *T. thermophila*, exceeding current eukaryotic benchmarks. This success likely stems from the organism’s unicellular nature, comprehensive life cycle sampling, advances in MS sensitivity, and an optimized proteogenomic pipeline (44). Despite this progress, ∼10% of predicted genes remain undetected, which is a common proteomic limitation attributable to: (i) transient expression during specific developmental or stress conditions; (ii) low-abundance proteins below MS detection thresholds; (iii) challenges in detecting hydrophobic membrane proteins; and (iv) potential gene prediction errors or non-functional gene models. In addition to enabling protein-level validation, our proteogenomic data also contribute to the refinement of gene models. One limitation of bottom-up shotgun proteomics is that it cannot directly verify whether the annotated start codon encodes methionine or confirm stop codons, which do not yield detectable peptides. However, the detection of peptides extending beyond annotated start or stop codons, or those spanning exon–intron junctions, can reveal structural inconsistencies in gene models. In this study, we identified 68 genes with likely incorrect start codons, 71 with misannotated stop codons, 110 with intron boundary errors, and 3 introns that appear to be misannotated coding regions.

Prior work on *T. thermophila* identified 2238 phosphorylation sites across 1008 proteins, linking phosphorylation to diverse processes such as transport and gene expression (97). Comparative analysis revealed 44 overlapping proteins between our dataset and the previously reported phosphoproteome, including the well-characterized phosphoprotein HHO1 (98), which was identified based on six phospho-threonine–containing peptides. We also identified phospho-serine modifications on histone H4 variants HHF1 and HHF2, and discovered potential novel phosphorylation sites at S140 of α-tubulin and T107 of β-tubulin. Furthermore, several intriguing novel acetylation events were identified in our dataset. Multiple ubiquitin-related proteins, including polyubiquitin, TTU3, and UBI4, were found to be acetylated. Although ubiquitin PTMs have been extensively studied, researches have primarily concentrated on phosphorylation, as exemplified by PINK1-mediated Ser65 phosphorylation (99), whereas acetylation of ubiquitin remains largely unexplored (100). Here, we provide the first *in vivo* evidence of ubiquitin acetylation in *T. thermophila*, laying the groundwork for future studies on its functional roles. In addition, acetylation of the ciliate-specific granule lattice proteins GRL1, GRL3, and GRL4 were detected. These proteins serve as scaffolds for the *Tetrahymena* dense-core granules, determine their crystalline morphology, and mediate rapid, stimulus-induced expansion during exocytosis (101). To our knowledge, this is the first report of acetylation on GRL proteins, suggesting that acetylation may regulate the assembly, stability, or function of these uniquely evolved non-membranous cellular structures.

Histone H3 and H4 methylation and acetylation have been well characterized in *T. thermophila*. Consistent with previous findings (102), our dataset confirmed acetylation at H3K14 (histone H3 lysine 14) and the characteristic methylation at H3K27 (histone H3 lysine 27; me1, me2, and me3). Variability in protein collection and analysis strategies may contribute to discrepancies in modification identification. In our dataset, several novel or less-characterized histone marks in *T. thermophila* were also identified. Acetylation at K122 was detected in H3 variants HHT1, HHT2, and HHT3, which has been reported in humans (103). Trimethylation at K122 was detected in HHT1 and HHT2. This lysine residue has also been reported to undergo crotonylation and ubiquitination in other eukaryotes (104, 105), highlighting its potential as a conserved epigenetic hotspot. Moreover, acetylation at K166 was uniquely identified in the HHT4 variants. Histone H4 variants (HHF1 and HHF2) exhibited acetylation at K91, a modification reported in yeast (106), as well as a potentially novel trimethylation at K59, although this was supported by only two modified peptides. In addition, malonylation at K53 was identified in H3 variants, representing a novel PTM in this context. Interestingly, H4 variants displayed a broader repertoire of modifications, including not only acetylation and trimethylation, but also phosphorylation, sumoylation, sulfation, palmitoylation, tyrosine nitration, malonylation, and crotonylation. This diversity underscores the potential complexity of histone regulation in ciliates, and suggests that *T. thermophila* may employ an expanded set of histone PTMs to modulate chromatin dynamics and fine-tune gene expression in response to developmental or environmental cues. PTMs of tubulins also merit attention. In *T. thermophila*, we identified a wide array of PTMs distributed across α-tubulin (ATU1; 21 types) and β-tubulin (BTU1; 22 types). The acetylation at the highly conserved K40 residue of α-tubulin was detected, which is a well-characterized modification involved in regulating microtubule stability, intracellular transport, and ciliary and flagellar function (107). In addition, previously unreported phosphorylation sites were identified at S140 of α-tubulin and T107 of β-tubulin. Most other modifications remain poorly characterized and may represent novel regulatory events deserving further investigation. Collectively, these PTMs fine-tune ribosomal activity, epigenetic regulation, cytoskeletal dynamics, and proteostasis to meet metabolic demands across life states. While this study highlights PTM-associated mechanisms, experimental validation of modification–function relationships remain essential.

In summary, the proteogenomic strategy employed in this study enabled a comprehensive reassessment of gene discovery in *T. thermophila*. The identified novel coding genes and extensive protein PTMs offer valuable resources for further mechanistic investigations into the life cycle of this model protozoan. These findings not only deepen our understanding of eukaryotic proteomic complexity but also underscore the utility of proteogenomics in unraveling the regulatory networks governing developmental transitions and environmental responses in unicellular systems.

## Conclusion

This study employed a comprehensive proteogenomic strategy to systematically reassess gene discovery of *T. thermophila*. By integrating high-resolution MS data spanning 10 life cycle states with the telomere-to-telomere MIC reference genome, we validated 90.9% of predicted protein-coding genes and identified 383 novel genes, thereby addressing critical gaps in the current gene catalog. Furthermore, systematic profiling of the protein PTM landscape revealed dynamic adaptations in modification networks that align with the functional demands of distinct developmental states. These findings not only underscore the efficacy of proteogenomics in assessing gene discovery in single-celled eukaryotes with complex life cycles but also establish a critical resource for investigating mechanisms of reproductive development and environmental adaptation in this model organism. Future research building on these reassessment results could prioritize functional validation of novel genes and PTM-mediated regulatory networks, advancing our understanding of conserved molecular mechanisms in eukaryotic evolution and their adaptive significance.

## Data Availability

The raw MS data, along with annotated MS/MS spectra for protein identifications supported by a single unique peptide and for all identified post-translationally modified peptides, have been deposited in the publicly accessible iProX database (http://www.iprox.org) under the identifier IPX0011857000. Any other data that support the results and conclusions of this study are available within the paper and its supplemental materials.

## Supplementary Data

This article contains supplemental data.

## Conflict of Interest

The authors declare that they do not have any conflicts of interest with the content of this article.

## Supplementary data

Supplementary Tables

Protein_Unique_Peptides.fa
