# Supplementary material for: Proteogenomic Reassessment Provides Novel Insight into the Life Cycle of Tetrahymenathermophila
Source: Mol Cell Proteomics. 2025 Sep 30;24(11):101081. doi: 10.1016/j.mcpro.2025.101081 (PMC12615304; doi:10.1016/j.mcpro.2025.101081)
Supplement: Supplementary Figures [file mmc3.docx]

**F_IG_. S1. Proteomic coverage of genome‐predicted coding genes.** Of the 26,742 genes predicted from the genome, 24,319 (90.9%) were experimentally detected by proteomic analysis.

**F_IG_. S2. Peptide‑match counts and sequence‑coverage distributions for the *T. thermophila* proteome by MS data.** *A,* Distribution of peptide segment lengths and corresponding number of identified proteins, and each identified protein is matched by an average of 300 peptides. *B,* Distribution of peptide number in each sample. The solid line traces the average number of peptides mapped to per protein in each sample. The peptides shown in panels A and B represent all matched peptides, not unique peptides. *C,* Distribution of sequence coverage among identified proteins. The average sequence coverage per proteins is 20.26% and a total of 567 proteins had coverage of 90-100%. *D,* Average sequence coverage across different samples.

**F_IG_. S3. Functional characterization of all proteins identified by proteomic analysis.** *A,* GO classification of identified proteins in the terms of biological process and molecular function. *B,* Functional classification based on KOG database. *C,* Predicted subcellular localization of identified proteins.

**F_IG_. S4. KOG functional classification of differential protein groups between different samples.** *A,* Vegetative growth (G) versus 3h starvation (S3). *B,* 15h starvation (S15) versus pair formation state (C2). *C,* Pair formation state (C2) versus new MAC differentiation state (C12).

**F_IG_. S5. Distribution of PSMs across functional annotation categories in each sample.**

**F_IG_. S6. Correlation analysis between proteomic and transcriptomic data for all identified proteins.** *A,* Venn diagram showing the overlap (17,586) between identified protein groups (18,486) and transcripts (24,381). *B,* Scatterplots comparing of transcript abundance (FPKM) and protein abundance (NSAF) across different samples (G, S3, C2, 3A1-27G, 3A1-27S, 3A1-80G, 3A1-80S). Pearson’s correlation coefficients (r) range from 0.3489 to 0.6304.

**F_IG_. S7. GO classification of identified PTM proteins.**

**F_IG_. S8. Genes involved in Cd and Hg metabolism in *T. thermophila*.** *A,* Schematic overview of *T. thermophila* genes putatively participating in Cd and Hg metabolism, grouped by predicted function: recognition, transport, binding and conversion. Gene IDs and their corresponding roles are indicated. *B,* Heatmap illustrating the relative expression (log₂FC) of Cd-metabolism-related genes in *T. thermophila* following exposure to CdCl₂ at concentrations corresponding to EC₅₀ (5.067 μg/mL) and EC₈₀ (5.534 μg/mL). Gene expression levels were normalized to untreated controls. *C,* Heatmap illustrating the relative expression (log₂FC) of Hg-metabolism-related genes in *T. thermophila* following exposure to HgCl₂ at concentrations corresponding to EC₅₀ (12.4 μg/mL) and EC₈₀ (16.82 μg/mL). Gene expression levels were normalized to untreated controls.

**F_IG_. S9. Length-dependent density profile of genome-predicted proteins, proteome-identified proteins, and newly identified proteins.** The density peak of the newly identified proteins is notably lower compared to the genome-predicted and proteome-identified protein groups.

**F_IG_. S10. Proteogenomic characterization of newly identified genes.** *A,* Length distribution of novel proteins. The average protein length is 69 amino acids. *B,* Distribution of PSM for all novel proteins. The average number of PSMs per novel protein is 66. *C,* GC content of the coding sequences encoding the predicted proteins in the database (DB), proteins identified by proteomic analysis and novel proteins. Novel genes exhibit a slightly higher GC content compared to the other two groups. *D,* Initiation Codon among novel genes, with ATG being the most frequently used.

**F_IG_. S11. Functional annotation and correlation analysis between transcriptome and proteome of novel genes.** *A,* Venn diagram showing that 347 of the 383 novel genes are supported by transcriptomic evidence. *B,* Scatterplots comparing transcript abundance (FPKM) versus protein abundance (NSAF) across seven states (G, S3, C2, 3A1-27G, 3A1-27S, 3A1-80G, 3A1-80S). Pearson’s correlation coefficients (r) for each condition are indicated on the plots. *C–D,* GO classification of newly identified genes in the terms of molecular function and cellular component.
